# Association between added sugar intake and dental caries in Yup’ik children using a novel hair biomarker

**DOI:** 10.1186/s12903-015-0101-z

**Published:** 2015-10-09

**Authors:** Donald L. Chi, Scarlett Hopkins, Diane O’Brien, Lloyd Mancl, Eliza Orr, Dane Lenaker

**Affiliations:** Department of Oral Health Sciences, University of Washington School of Dentistry, Box 357475, Seattle, WA 98195 USA; University of Alaska Fairbanks, Center for Alaska Native Health Research, Fairbanks, AK USA; Yukon Kuskokwim Health Corporation, Dentistry Department, Bethel, AK USA

**Keywords:** Added sugars, Dietary biomarkers, Sugar-sweetened beverages, Dental caries, Alaska Native children, Oral health disparities, Oral health interventions

## Abstract

**Background:**

Dental caries (tooth decay) is a significant public health problem in Alaska Native children. Dietary added sugars are considered one of the main risk factors. In this cross-sectional pilot study, we used a validated hair-based biomarker to measure added sugar intake in Alaska Native Yup’ik children ages 6–17 years (N = 51). We hypothesized that added sugar intake would be positively associated with tooth decay.

**Methods:**

A 66-item parent survey was administered, a hair sample was collected from each child, and a dental exam was conducted. Added sugar intake (grams/day) was measured from hair samples using a linear combination of carbon and nitrogen ratios. We used linear and log-linear regression models with robust standard errors to test our hypothesis that children with higher added sugar intake would have a higher proportion of carious tooth surfaces.

**Results:**

The mean proportion of carious tooth surfaces was 30.8 % (standard deviation: 23.2 %). Hair biomarker-based added sugar intake was associated with absolute (6.4 %; 95 % CI: 1.2 %, 11.6 %; *P* = .02) and relative increases in the proportion of carious tooth surfaces (24.2 %; 95 % CI: 10.6 %, 39.4 %; *P* < .01). There were no associations between self-reported measures of sugar-sweetened food and beverage intake and tooth decay.

**Conclusions:**

Added sugar intake as assessed by hair biomarker was significantly and positively associated with tooth decay in our sample of Yup’ik children. Self-reported dietary measures were not associated tooth decay. Most added sugars were from sugar-sweetened fruit drinks consumed at home. Future dietary interventions aimed at improving the oral health of Alaska Native children should consider use of objective biomarkers to assess and measure changes in home-based added sugar intake, particularly sugar-sweetened fruit drinks.

## Background

The Alaska Natives Commission released a Final Report in 1994 that included an assessment of the physical health of Alaska Natives [1]. The Commission cited pediatric dental caries as one of the “new health problems facing Alaska’s Native people”. Twenty years later, the pediatric caries epidemic in Alaska Native communities has not abated. Dental caries is the most common disease worldwide [2]. Alaska Native children are disproportionately more likely than other children to experience tooth decay, with prevalence rates ranging from 87 to 98 % [3, 4]. For example, Alaska Native and American Indian children ages 2 to 5 years had 4.13 decayed, missing, or filled primary teeth (dmft) compared to 1.17 dmft for U.S. children ages 2 to 5 years [4, 5]. When left untreated, dental caries can lead to pain, infection, missed school days, hospitalization, poor quality-of-life, and in rare cases death [6–9]. Furthermore, childhood tooth decay is one of the strongest predictors of tooth decay in adulthood [10], underscoring the importance of preventing dental disease in children.

There are three behaviors involved in caries prevention. The first is regular dental visits, which give dentists an opportunity to assess a child’s risk for developing caries, provide risk-based anticipatory guidance, identify strategies to mitigate risk factors, and deliver preventive care (*e.g.*, topical fluoride treatments, pit-and-fissure sealants). In an observational study, ≥4 fluoride varnish treatments provided during medical well baby visits reduced tooth decay rates in American Indian children [11]. Dental care is limited in many rural Alaska Native communities, which are geographically isolated and not connected by roads to larger population centers.

The second is exposure to topical fluorides. Fluoridated community water is the most cost-effective population-level dental caries prevention strategy, but most Alaska Native communities do not have piped-in water, which reduces the scalability and feasibility of water fluoridation [3, 12]. There are also local concerns in Alaska Native communities related to Hooper Bay in which a water supply was inadvertently hyperfluoridated, leading to the only documented death related to fluoridation [13]. Most Alaska Native children do not receive fluoride supplementation as recommended for children without access to fluoridated water and do not brush their teeth regularly with fluoridated toothpaste [14, 15].

The third is limiting carbohydrate intake. Dietary sources of caries-causing carbohydrates include sugar-sweetened beverages, juices, candies, crackers, and chips, and to a lesser extent breads, rice, and pastas [16–18]. Sugar-sweetened beverages, which contain high concentrations of added sugars, are a major source of dietary carbohydrates in U.S. children and adolescents [19, 20]. Over 25 % of U.S. children consume ≥1 non-diet sodas/week and 16 % consume other sugar-sweetened beverages [21]. A recent study of Alaska Native Yup’ik individuals found flavored drinks (like sugar-sweetened fruit drinks) and soda were among the most commonly consumed foods [22]. Numerous studies indicate a positive relationship between sugar-sweetened beverages and tooth decay in children, particularly in low-income children [23, 24].

The American Academy of Pediatrics policy statement called for research to identify public health strategies to address the caries epidemic in Alaska Native communities, but few oral health interventions have been tested [25, 26]. Based on the unresolved tooth decay epidemic among low-income children and minorities in the U.S., the president of the American Dental Association has called for additional research focusing specifically on the oral health effects of added sugar [27]. Anecdotal evidence from health providers and parents in Alaska Native communities indicate carbohydrate intake, particularly added sugars from beverages, as an important intervention target for children.

Public health interventions aimed at reducing carbohydrate intake require valid and objective measures. Self-reported measures of dietary intake are susceptible to high levels of error and bias, making it difficult to detect dietary associations with health outcomes [28, 29]. Sugar intake is particularly subject to misreporting [30, 31]. This problem has created interest in several new approaches to measuring sugar intake using objective biomarkers [32–37]. One approach measures the carbon isotope ratio, which is elevated in corn and sugar cane, the source of 75 % of sugars consumed in the U.S. [38–40] and nearly all of the sugars consumed in rural Alaska Native communities. Previous work indicates a linear combination of red blood cell carbon and nitrogen isotope ratios is strongly predictive of total sugar, added sugar, and sugar-sweetened beverage intake in an Alaska Native population, validated against repeated 24-h recalls, the gold standard of dietary self-report data [41]. This objective measure has been used to assess associations between sugar intake and risk factors for chronic disease in the same Alaska Native population [42], but has not yet been used with children or in studies of oral health.

In this pilot study, we evaluated the feasibility of collecting hair samples from Yup’ik children and tested the association between our hair biomarker-based measure of added sugar intake and tooth decay. We hypothesized there would be a positive relationship between our biomarker measure of added sugar and tooth decay. This investigation is the first step in evaluating how an objective added sugar biomarker can be used in future public health interventions aimed at preventing tooth decay in Alaska Native children.

## Methods

### Study location

The study was conducted in Bethel, Alaska, the largest city in the Yukon-Kuskokwim (YK) Delta. Bethel is located 400 miles west of Anchorage and is the regional hub for 58 villages. The Bethel Census Area has about 18,000 residents and 81.8 % of the population is Alaska Native, the majority of whom are of Yup’ik descent [43]. Thirty-six percent of the population is under age 18 years, the per capita income is $19,055, and 21.8 % of the population lives below poverty [43].

### Study design and population

We recruited a convenience sample of Alaska Native children in 2014 who sought dental care at the Yukon-Kuskokwim Health Corporation (YKHC) Dental Clinic in Bethel. This was a pilot study and there were no a priori sample size calculations. The recruitment goal was 50 children and adolescents but we had approval to enroll up to 60 participants. Inclusion criteria were: 1) ages 6 to 17 years; 2) self-identified as Yup’ik; 3) ≥2 cm of hair length (to allow for collection of an adequate hair sample); and 4) assent from the minor and parental consent. Children received a $10 grocery store gift card and parents received a $20 grocery store gift card. Both received a toothbrush, toothpaste, and floss. The study was approved by the YKHC Board of Directors, the University of Washington Institutional Review Board (IRB), and the University of Alaska Fairbanks IRB.

### Study procedures

A clinic receptionist approached and pre-screened potential participants. For interested families, a study staff member verified inclusion criteria, explained the study and procedures, and answered questions. After obtaining consent from the parent and assent from the child, there was a three-step study process.

First, a 66-item survey was administered verbally by a Yup’ik member of the research team in either English or Yup’ik based on parent preference. The survey was developed specifically for this study and we ensured that all questions were culturally appropriate by pre-testing the survey with a Yup’ik parent. We asked questions on child and family demographics (*e.g.*, age, sex, residence, household income) and child oral health behaviors (*e.g.*, sugar-sweetened food and beverage intake, toothbrushing, fluoride access). The food and beverage intake questions were adapted from the Beverage and Snack Questionnaire previously developed for use in school settings and asked about intake frequency of popular items consumed by children as well as place in which the items were consumed [44]. If a parent was unable to answer a question, the child was asked to provide a response.

Second, we cut about 20 strands of hair from the back of each child’s head. Each hair sample was taped so the 2 cm segment of hair closest to the scalp could undergo isotope analyses. One centimeter of hair corresponds to approximately 1 month of growth and reflects added sugar intake from the previous 1–2 months. Each hair sample was stored in a plastic bag and transported for processing and analysis. The 1 cm section of the hair sample most proximal to the scalp was cleaned, prepared, and analyzed for carbon and nitrogen isotope ratios using continuous flow isotope ratio mass spectrometry at the Alaska Stable Isotope Facility [43]. Added sugar intake was generated from δ^13^C and δ^15^N using previously estimated coefficients for the Yup’ik population [45–47].

Third, each participant received a tooth surface-level dental exam based on World Health Organization criteria [48]. A board-certified pediatric dentist conducted the exams in a private dental operatory. The teeth were cleaned with a dry toothbrush and dried with gauze and air, and exams were completed using a mouth mirror and light. No dental explorers were used. Visible tooth surface were classified as present and sound, decayed (non-cavitated or cavitated), missing due to caries, or restored (filled or crowned). Non-cavitated carious lesions were assessed visually consistent with published recommendations [49]. To assess intrarater reliability, 10 % of the participants were randomly selected for a second exam (ICC = 0.92). A random number generator was used to select participants for repeat exams. Clinical findings were summarized to parents. Children requiring treatment were referred for follow-up care.

### Variables

The outcome variable was the proportion of carious tooth surfaces, measured as the number of decayed (non-cavitated and cavitated), missing, and restored primary and permanent tooth surfaces divided by the total number of tooth surfaces present. Added sugar intake (g/day) was a continuous predictor variable measured using the hair sample. Child’s age was modeled as a confounder based on previous studies [21, 50]. There were no other model covariates.

### Data analyses

We generated demographic statistics and summarized survey data on oral health behaviors. Next, we evaluated distributional assumptions for the outcome. Because the mean and median proportions of carious tooth surfaces were similar and the distributions were not skewed, we used linear regression analyses to assess the association between added sugar intake and tooth decay. To facilitate the interpretation of the regression models, added sugar intake/day was divided by 40 g, corresponding to the difference in the proportion of carious tooth surfaces associated with a 40-gram increase in added sugar intake (*i.e.*, amount of sugar in a 12-ounce soda). We observed a quadratic association between age and the outcome, with a minimum observed around age 12 years (corresponding to exfoliation of primary teeth and eruption of permanent teeth). Thus, we centered age at 12 years and included a quadratic effect for age. To confirm the linear regression results, we used log-linear regression models and included an offset equal to the logarithm of the total number of surfaces present. The log-linear regression models were fit using generalized estimating equations with robust standard errors [51]. As part of the sensitivity analyses, we ran our regression models with and without non-cavitated lesions as part of the outcome. The results were identical. Therefore, we reported findings from models including non-cavitated lesions. All analyses were completed using statistical software R version 3.0 (R Foundation for Statistical Computing, Vienna, Austria) and SAS version 9.3 (SAS Institute, Cary, NC).

## Results

### Demographic statistics

We enrolled 51 participants. The study population was 50 % female and the mean age was 10.8 years (standard deviation: 3.3 years). Forty-three percent lived in Bethel and the remaining 57 % children lived elsewhere in the YK Delta. About 30 % of children lived in families with a total annual household income < $20,000, 17.6 % had an income $20,000-$29,999, 21.6 % had an income $30,000-$49,999, and 19.6 % had an income > $49,999.

### Oral health behaviors

Regarding beverage consumption data collected via parent survey, 49 % of children were reported to consume sugar-sweetened fruit drinks (*e.g.*, Tang, Kool-Aide) 2–3 times/day and 15.7 % reported consuming sugar-sweetened beverages ≥4 times/day at home. Nearly 14 % consumed soda 2–3 times/day, 43.1 % consumed sodas 1–4 times/week, and 33.3 % never consumed soda. Over 45 % consumed 100 % juice (*e.g.*, orange, apple, grape) and 65 % of children drank water.

In terms of home-based snack and food consumption, 70.6 % of children consumed sweets (*e.g.*, candies, Sour Patch Kids, gummy bears, Life Savers) and 78 % consumed salty snacks (*e.g.*, chips, Chex Mix, Gold Fish) at least once/week. Less than 30 % of children had a serving of vegetables at least once/day and 15.7 % never ate vegetables at home. Less than 14 % had a serving of fruit at least once/day and 17.6 % never ate fruit.

Nearly 30 % of parents reported their child brushed their teeth more than once/day, 37.3 % brushed once/day, and 29.4 % brushed 1–3 times/week. All parents reported their child used commercially available toothpaste (*e.g.*, Crest, Aim, Colgate), but 52.9 % did not know whether the toothpaste contained fluoride. Over 94 % reported their child had never been prescribed fluoride by a dentist or physician and 23.5 % of parents reported their child had not received a fluoride treatment in the past year from a dentist.

### Dental caries and added sugar intake

**Fig. 1:**
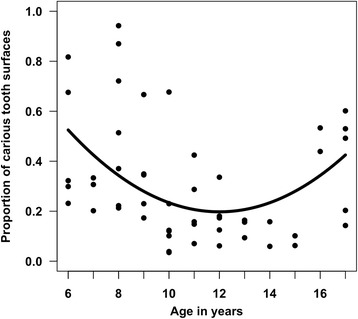
Relationship between age and proportion of carious tooth surfaces

**Fig. 2:**
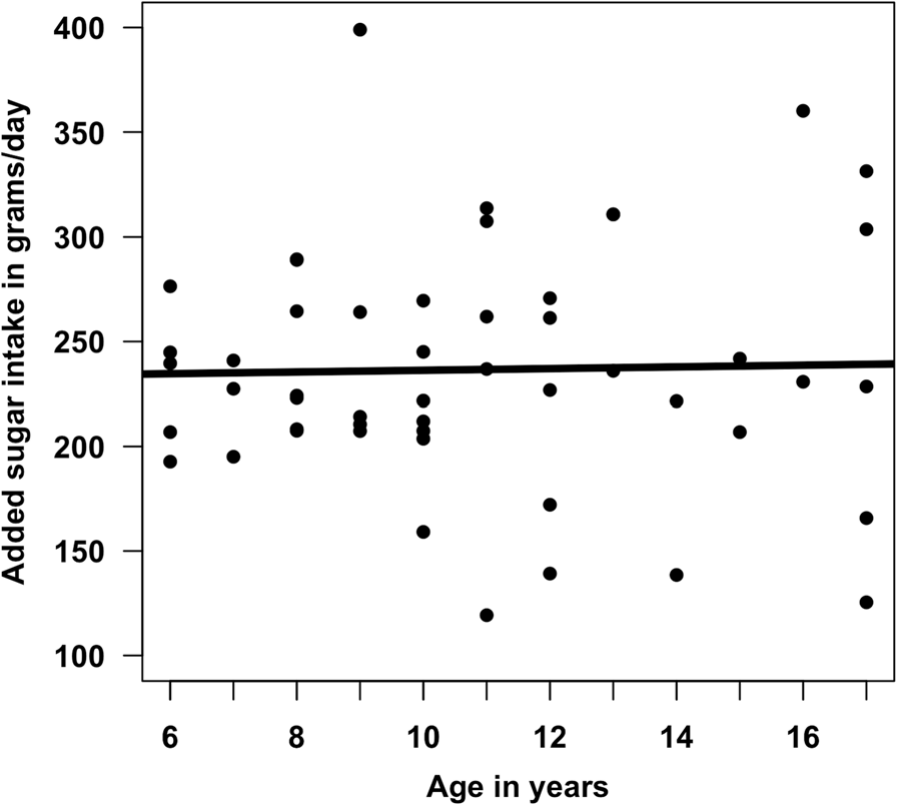
Relationship between age and added sugar intake (g/day)

**Fig 3:**
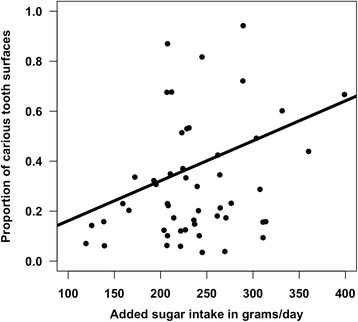
Relationship between added sugar intake and proportion of carious tooth surfaces

The mean proportion of carious tooth surfaces was 30.8 % (standard deviation: 23 %; range: 3 % to 94 %). Most carious tooth surfaces were filled (mean number of filled surfaces: 12.3; standard deviation: 12.7), followed by non-cavitated decayed surfaces (mean: 9.0; standard deviation: 8.3), crowned surfaces (mean: 5.8; standard deviation: 10.6), cavitated decayed surfaces (mean: 4.3; standard deviation: 7.1), and missing surfaces (mean: 1.6; standard deviation: 4.6). Caries rates followed a U-shaped curve, decreasing from ages 6–12 years and then increasing from ages 13–17 years (Fig. 1). The mean added sugar intake was 193 g/day (standard deviation: 43.6; range: 105.6 to 324.3 g/day). Added sugar intake was constant across age (Fig. 2). There was a positive, significant relationship between our biomarker measure of added sugar intake and dental caries (Fig. 3). There were no significant associations between self-reported measures of sugary foods and beverage intake and tooth decay.

### Regression models

In the age-adjusted linear regression model, a 40-gram/day increase in added sugar intake was associated with a 6.4 % absolute increase in the proportion of carious tooth surfaces (95 % confidence interval: 1.2 %,11.6 %; *P* = .02). In the log-linear regression model, a 40 g/day increase in added sugar intake was associated with a 24.2 % relative increase in the proportion of carious tooth surfaces (95 % CI: 10.6 %, 39.4 %; *P* < .01). Findings were similar when the analyses were restricted to children with ≥15 primary tooth surfaces and children with only permanent teeth.

## Discussion

We found a positive association between biomarker-assessed added sugar intake and dental caries in Yup’ik children and adolescents, but not with self-reported intake of sugar-sweetened foods and beverages. The association between added sugar intake and caries is consistent with previous studies [52]. Participants also reported consuming high level of sugar-sweetened fruit drinks (*e.g.*, Tang, Kool-Aide) relative to soda and other sources of dietary sugar. Most of these beverages were consumed at home. These findings are consistent with recent studies showing high intakes of sugar-sweetened fruit drinks and soda by Yup’ik adults [22], and suggest that public health efforts focusing on reducing sugar-sweetened fruit drinks could significantly improve children’s oral health in this population.

This is the first study to use an objective biomarker of sugar intake to evaluate the relationship between dietary sugar intake and caries in children. Biomarkers of sugar intake have only been developed and validated within the last 10 years, and several of them, specifically measures based on blood [32] or 24-h urine collection [35], would be difficult to use in studies involving children. The hair-based biomarker used in this study was collected easily and non-invasively, and detected significant associations with tooth decay despite the small sample size of the study and despite the fact that the biomarker was calibrated in Yup’ik adolescents and adults ages 14–79 years [45–47, 53]. This is a promising finding that warrants future research focusing specifically on children of Yup’ik descent.

The stable isotope biomarker indicated that children and adolescents in this study consumed an average of 193 g of added sugar/day (equivalent to the amount of sugar in 5, 12-ounce sodas). The American Heart Association recommends no more than three teaspoons of sugar (or 12 grams) for children each day [54]. Study participants consumed 16 times the maximum daily added sugar intake recommended for children. We interpret this value with some caution, because as noted above, the biomarker was calibrated specifically in Yup’ik adolescents and adults. However, it is clear from this study that added sugar intake is very high and is contributing significantly to dental disease. Based on our survey data, sugar-sweetened fruit drinks are likely to be the main source of added sugars among Yup’ik children, with most of these beverages consumed at home.

There is a need for home-based interventions that reduce added sugar intake to help prevent tooth decay as well as other systemic diseases linked to sugar like obesity, diabetes, hypertension, and cardiovascular disease [55–58]. Reducing sugar-sweetened beverage intake among Alaska Native children is likely to require culturally relevant public health interventions that educate communities on the high amount of sugar found in common sugar-sweetened beverages and increase access to acceptable alternatives like water or drinks with non-nutritive sweeteners [26]. Community-centered educational interventions could be implemented within stores, homes, and clinical settings [59, 60].

Yup’ik children and their families were enthusiastic about participating in this study, which we attribute to three factors. First, our study team involved a co-investigator who grew up in the YK Delta and is fluent in Yup’ik, which made parents and children comfortable and gave potential participants the opportunity to ask questions in their native language. We achieved our recruitment goals in one-third the anticipated time period (about 1 week to recruit 50 participants). Second, the study took place in a dental clinic, which did not require participants to travel specifically for the study. Third, oral health was highly salient to parents. Most parents described their own experiences with dental disease and were interested in ways to prevent tooth decay. In addition, we encountered no resistance from children in providing a hair sample, presumably because the amount required was minimal and hair was collected from a non-noticeable location on the head. Collectively, these findings indicate hair-based oral health studies are feasible in the YK Delta.

In addition to targeting sugar-sweetened beverage intake, future interventions could also identify cost-effective and scalable ways to improve access to preventive care. Alaska Native communities are small and geographically isolated, which make it possible to test innovative prevention strategies. One example is a home visitor or school-based program in which trained personnel from the community brush young children’s teeth with fluoride toothpaste and lead group toothbrushing activities with older children and adolescents [61]. This would address the irregular oral hygiene behaviors identified from our survey data. Additional research is needed to identify the feasibility and acceptability of such community-based oral health interventions.

Our study has public health, policy, research, and clinical significance. The public health significance of our study is that sugar-sweetened beverages in Alaska Native communities lead to diseases that place demands on a fragile health care system, which calls for community-based interventions aimed at reducing sugar-sweetened beverage intake. In addition to community-based interventions, upstream changes to the U.S. Supplemental Nutrition Assistance Program (SNAP), Women, Infants, and Children (WIC) Program, and Postal Bypass Program may be needed to reduce sugar-sweetened beverage purchases and eliminate subsidies for transport of unhealthy beverages to communities in rural Alaska. Although beverage taxes are likely to reduce demand for sugar-sweetened beverages [62], there is likely to be strong local resistance as demonstrated by the Bethel Finance Committee’s decision to unanimously oppose a $0.08/ounce sugar sweetened beverage tax in January 2013. Our biomarker-based measure of added sugar overcomes costs associated with self-reported measures of dietary sugars as well as the potential for individuals to underreport sugar intake. In fact, we found no statistically significant relationships between survey-based sugar-sweetened food and beverage intake and caries, highlighting limitations associated with self-reported dietary measures. Biomarkers could be used as surveillance tools to identify communities with the greatest need for public health interventions and to measure behavioral changes associated with implemented interventions.

There were four main study limitations. First, our analyses were cross-sectional, whereas the relationship between added sugar intake and dental caries is dynamic and modified by exposure to fluorides [63]. Second, the hair biomarker was specifically validated in a Yup’ik population ages 14–79 years [46], which does not cover the younger age range of participants in our study. However, added sugar intake was constant across age, which increases the likelihood that our biomarker accurately measures added sugar intake in younger children. Third, we recruited a small convenience sample of Alaska Native children seeking dental care, which is the population to which we can generalize our findings. Future studies should enroll a larger, community-based sample of study participants, to allow for a more comprehensive analysis of the risk factors associated with tooth decay in Yup’ik children. Fourth, we did not conduct a reliability assessment of survey items. However, none of our primary variables of interest were obtained through survey. Future research should assess the reliability and validity of oral health survey items in regards to Alaska Native populations.

## Conclusions

The tooth decay epidemic in Alaska Native communities underscores the importance of community-centered, prevention-oriented interventions aimed at improving health behaviors like reducing sugar-sweetened beverage intake. Preventive efforts should target the deleterious effects of added sugars by providing viable alternatives to sugar-sweetened beverages and making it easier for individuals to access fluorides and other types of preventive care that helps to control tooth decay. Future interventions should focus on educating communities about added sugars through stores, homes, and clinical settings, empowering parents and families to reinforce children’s exposure to fluoride through daily toothbrushing, and providing individuals with regular preventive care. Efforts to improve diet are likely to address the disease burden associated with pediatric tooth decay as well as reduce other childhood diseases linked to added sugars and sugar-sweetened beverages.
